# Comparing the efficiency of living and non-living macroalgae biomass in removing classical and emergent contaminants from complex multi-element mixtures

**DOI:** 10.1007/s00449-026-03292-z

**Published:** 2026-02-25

**Authors:** Jéssica Jacinto, Daniela Tavares, Nicole Ferreira, Thainara Viana, João Pinto, Nuno Lapa, Eduarda Pereira, Bruno Henriques

**Affiliations:** 1 1LAQV-REQUIMTE-Associated Laboratory for Green Chemistry, Department of Chemistry, Campus de Santiago, University ofAveiro, Aveiro, 3810-193 Portugal; 2 LAQV-REQUIMTE, Department of Chemistry, NOVA School of Science and Technology, NOVA University Lisbon, Caparica, 3rd Floor, 2829-516 Portugal

**Keywords:** Macroalgae, Biosorption, Bioconcentration, Rare earth elements, Potentially toxic elements, Complex mixtures

## Abstract

Although macroalgae are promising biosorbents for the removal of various contaminants, their effectiveness in complex mixtures requires comprehensive comparative evaluation under multi-contaminant conditions. The ability of living and non-living *Ulva lactuca* and *Gracilaria gracilis* to simultaneously uptake Rare Earth Elements (REEs) (Y, La, Nd, Eu, Gd, Dy) and classical contaminants (Hg, Cd, Pb, As) from equimolar mixtures was compared. Batch sorption experiments were conducted for 72 h under optimised conditions of salinity (10) and pH (7.8), in which 5 g of living biomass and an equivalent non-living biomass (0.60–0.85 g) were exposed to contaminated seawater (1 L) under constant stirring (800 rpm). The living biomass exhibited high removal rates (> 80%) for REEs, Hg, and Pb, while for As and Cd, lower removals were achieved. Non-living biomass showed significantly lower removal ability for REE (generally < 40%). These findings suggest a two-step approach, exploiting, first, non-living biomass to remove common contaminants and, after, living biomass for bioaccumulating REEs, which could later be recovered for reuse.

**Supplementary Information:**

The online version contains supplementary material available at 10.1007/s00449-026-03292-z.

## Introduction

Water pollution, climate change and the overexploitation of water resources are causing widespread water stress [57]. Rapid population growth has intensified pressure on water resources due to the expansion of the agricultural and industrial sectors, increasing the volumes of waste entering the water bodies [18].

Mercury (Hg) holds a prominent position (3rd) in the Agency for Toxic Substances and Disease Registry (ATSDR) list of priority pollutants due to its high frequency and persistence [1]. Lead (Pb), Arsenic (As), and Cadmium (Cd) are also classified as priority substances [30] since, unlike elements such as Copper (Cu), Zinc (Zn), Selenium (Se), and Iron (Fe), which play crucial biochemical roles, these are non-essential elements presenting toxicity even at low concentrations [19]. In addition, emerging contaminants such as Rare Earth Elements (REEs) are posing new challenges for water quality [45]. Anthropogenic enrichment with REEs has been reported in natural environments. Water samples from the Han River in South Korea showed positive Samarium (Sm), Lanthanum (La) and Gadolinium (Gd) anomalies [51]. REEs as emergent contaminants have, therefore, received increased attention, and studies have shown their negative impacts on aquatic organisms and human health [2, 8, 40].

Conventional methods to remove contaminants from water include adsorption, chemical precipitation, ion exchange, membrane filtration and reverse osmosis [59]. These pose drawbacks such as operational and maintenance high costs, showing inefficiency or generating hazardous wastes [31, 33]. Therefore, biosorption gained position as an economical, simple, and more environmentally friendly alternative [26, 43]. The underlying mechanisms involve either the interaction between non-living biomass and the contaminant, in a metabolically independent way, or with living biomass, in which bioaccumulation is involved with the active transport of elements into the interior of the organism’s cells [58]. Different chemical phenomena may occur, such as surface complexation, ion exchange, electrostatic interactions, or others with active functional groups [25, 49].

Different biosorbents have been explored, such as plant biomass [44], microorganisms [53], biopolymers [52], and agro-industrial wastes [37]. Among these, macroalgae are reported to bind classic contaminants, such as metals (Hg, Cd, Pb), and emergent contaminants, such as REEs [17]. However, their application requires further knowledge under contamination scenarios such as complex mixtures containing different types of contaminants (competing ions) [11]. Moreover, previous studies [32, 38, 39] have advocated that using living biomass is the most effective approach rather than using non-living biomass to remove Hg and other metals.

Therefore, the present study focused on a direct comparison between the removal efficiencies of living and non-living biomass of marine macroalgae in a complex mixture of As, Cd, Hg, Pb, yttrium (Y), La, europium (Eu), Gd, neodymium (Nd) and dysprosium (Dy) intended to cover wastewater from various sources where these elements could be extracted, such as industrial, mining, electronic or urban wastes [7, 50]. Moreover, the selected elements are utilised in a wide range of relevant electronic equipment and renewable energy technologies [4]. The novelty of the research consists, therefore, in the direct comparison between the living and non-living forms of the explored biosorbents–macroalgae (*Ulva lactuca* and *Gracilaria gracilis*)—in terms of their removal efficiency and selectivity in a mixture of complex contaminants. To the best of the authors’ knowledge, no other study compared this duality towards the sorption of REEs in complex mixtures for the forms of biosorbents selected.

## Material and methods

### Macroalgae collection and living and non-living biomass preparation

Macroalgae were collected at the Aveiro estuary (Portugal, 40º 34′35.1″N, 8º45′08.1″W), transported to the laboratory in isothermal plastic bags containing estuary water, and then rinsed with tap water to remove debris and epibionts. Samples were immediately freeze-dried for further quantification of the inherent metal concentrations (baseline). Table 1.

**Fig. 1:**
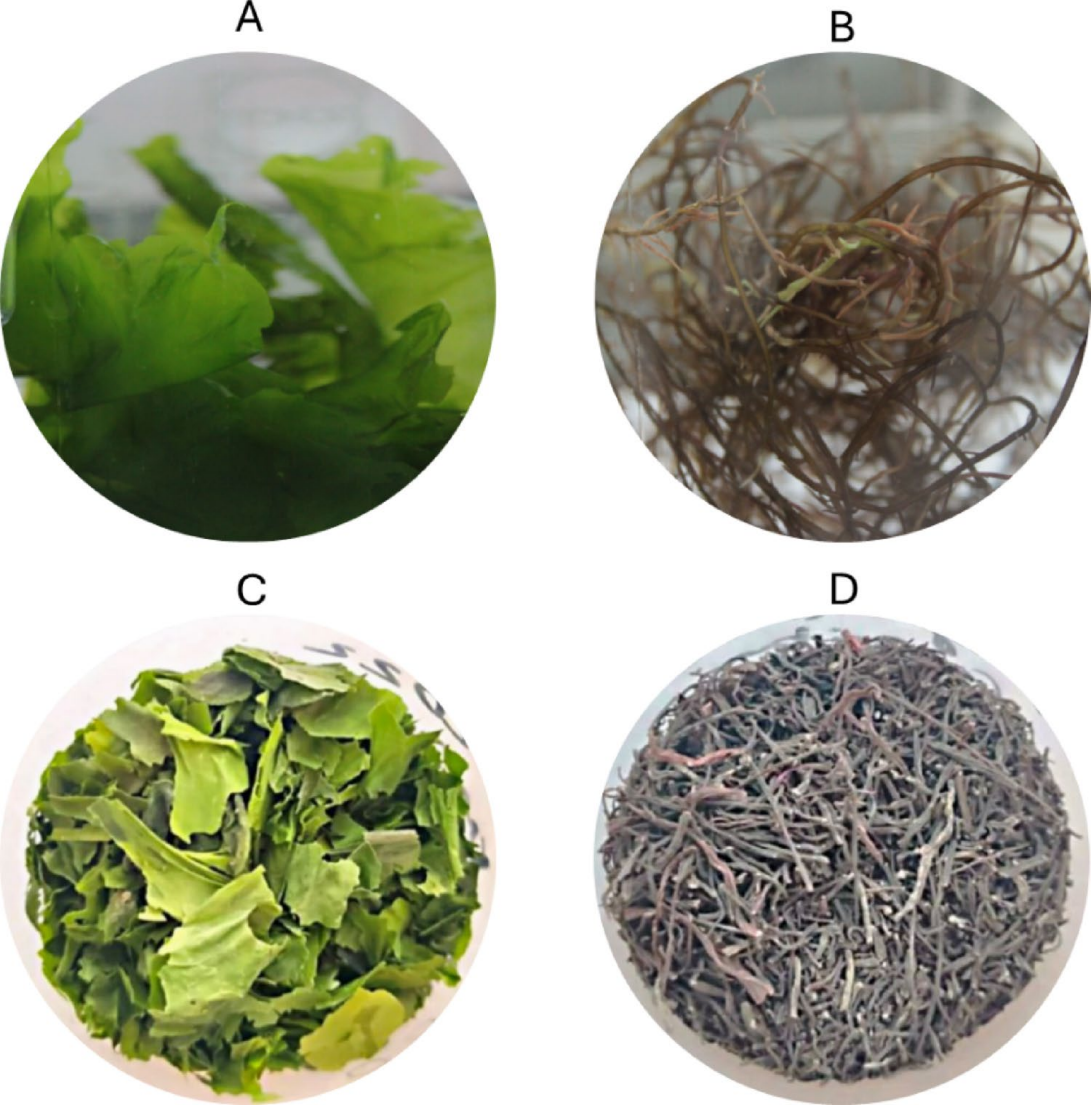
Living biomass of *Ulva lactuca* (**A**) and *Gracilaria gracilis* (**B**), and non-living biomass of *U. lactuca* (**C**) *and G. gracilis* (**D**)

Until the beginning of the experiments, macroalgae were maintained in 30 L clear glass aquariums equipped with air pumps and filled with filtered seawater, under natural light with a photoperiod of 12 h, at room temperature (20 ± 2 °C), without nutrient supply for prior acclimatisation. The dry/fresh weight ratio was assessed by gravimetric analysis [21, 34]. Briefly, fresh macroalgae samples (n = 20) were oven-dried at 40 °C for 48 h until constant weight, cooled in a desiccator, and reweighed. This ratio was used to calculate the equivalent dry biomass for the non-living experiments. To prepare the living biomass (metabolically active thalli), several pieces were cut from the thallus of similar sizes. Approximately 5 g of fresh macroalgae was added to each Schott flask with the mixture of contaminants. No further pre-treatments were done on the macroalgae biomass. For the preparation of the non-living biomass, several pieces of the two macroalgae species were placed in an oven at 40 °C for 2 days, before being milled using a domestic coffee grinder. Figure 1 presents the two forms of the biosorbent used in this experimental assay for both species of the macroalgae.

### Reagents

All laboratory material was washed and filled with 25% (v v^-1^) HNO_3_ (Merck, supra-pure, 65% m m^-1^) for at least 24 h and then rinsed with ultrapure water (Milli-Q, 18 MΩ cm^-1^). Single-element standard solutions of REEs in HNO_3_ (1–7%) were obtained from certified suppliers: Alfa Aesar (Y, Dy), Inorganic Ventures (Nd), Plasma Cal (La, Eu), and Sigma Aldrich (Gd). The certified standard solutions of mercury nitrate (Hg(HNO_3_)_2_: 1001 ± 2 mg L^− 1^), arsenic acid (H_3_AsO_4_: 1000 ± 2 mg L^− 1^), cadmium nitrate (Cd(HNO_3_)_2_: 1000 ± 2 mg L^− 1^), and lead nitrate (Pb(HNO_3_)_2_: 1000 ± 2 mg L^− 1^) were purchased from Merck.

### Seawater collection and preparation of contaminated saline solutions

Seawater was collected at Barra beach (Portugal, 40°33′N, 8°46′W), filtered through 0.45 μm Millipore membranes and kept in the dark at 4 °C. A physicochemical characterisation including pH, conductivity, salinity, and major and minor elements has been reported by Henriques et al. [29].

The contaminated saline solutions were prepared by dilution of the filtered seawater with ultrapure water to a final salinity of 10. This salinity has been chosen since it is a common value in estuaries that are receptacles of industrial discharges containing these pollutants [29]. Previous studies have demonstrated that, at this salinity, in a range from 10 to 35, the highest removal percentages are achieved [13]. Adequate volumes of REEs standard solutions were employed to spike the saline water, ensuring equimolar concentrations of the elements for a more accurate comparison. The initial concentrations of all elements in the solution, expressed in µg L^− 1^, are presented in Table 1. The real concentrations of these elements in the prepared mixtures were between 54 and 323 µg L^− 1^ and were selected considering reported values for contaminated waters [14]. The conditions of the study were fixed as follows: salinity 10 and pH 7.8.

### Quantification of elements in solution

Element concentrations in the saline solution were determined using Inductively Coupled Plasma Mass Spectrometry (ICP-MS) on a Thermo Scientific X Series Quadrupole instrument. Calibration curves were built with several multi-element standards, previously diluted from a certified standard in HNO_3_ 1%, with concentrations ranging from 0 to 10 μg L^− 1^. The Limit of Quantification (LOQ) was assumed to be the concentration of the lowest standard used in the calibration curve, while 1/3 of this value corresponded to the Limit of Detection (LOD) [22]. The limits of quantification were 2 µg L^− 1^ for As, 0.1 µg L^− 1^ for Cd, 0.1 µg L^− 1^ for Pb, 0.2 µg L^− 1^ for Y, 0.02 µg L^− 1^ for La, Nd and 0.01 µg L^− 1^ for Eu, Gd, and Dy, with an acceptable relative standard deviation among replicates (≤ 10%). To avoid matrix interferences, all samples were diluted 10 times with HNO_3_ 2% before quantification [24].

Mercury quantification in water samples was performed by cold vapour atomic fluorescence spectroscopy (CV-AFS) in a PSA 10.025 Millennium Merlin Hg analyser and using SnCl_2_ (2% m v^-1^ in HCl 10% v v^-1^) as a reducing agent [20]. In the range of operational conditions used, the LOQ was 0.021 mg L^− 1^ (n = 20), with an acceptable relative standard deviation among replicates (≤ 5%).

### Batch sorption assays in multi-element solutions with living and non-living biomass

Batch sorption experiments were performed in Schott Duran^®^ glass flasks (1 L; 253 mm overall height and 93 mm outer diameter), at room temperature (20 ± 2 °C), by contacting 5 g of fresh living biomass with 1 L of saline solutions spiked with the elements under study, at constant stirring (800 rpm, with a magnetic stirrer of 40 mm × 8 mm). The amount of biomass used was previously optimised (data not shown). Parallel batch sorption assays were performed with the same experimental design used for living biomass. The amount of biosorbent used was the equivalent in dry weight to the mass used in sorption assays with living biomass, which was calculated based on the water content of each species (88% for *Gracilaria gracilis* and 83% for *Ulva lactuca*) [21, 34]. For *Ulva lactuca* and *Gracilaria gracilis*, 0.85 and 0.60 g of dried biomass were used, respectively. After the addition of the macroalgae to the contaminated seawater, samples (ca. 10 mL) were collected at pre-defined times until 72 h (1, 3, 6, 9, 24, 48, and 72 h) and then acidified to pH < 2 with HNO_3_ (65% m/m). The samples collected from the experiments with non-living biomass were centrifuged to separate the biomass from the solution, prior to analysis. Quality control was consistently maintained throughout the experiment by running duplicate flasks for each treatment (biological replicates; n = 2), and two duplicate samples were analysed from each replicate flask (analytical replicates). Each sample was analysed at least 3 times with an acceptable relative standard deviation among replicates of ≤ 5% for Hg and ≤ 10% for the other elements under study. Controls, defined as saline water spiked with the elements under study, in the absence of macroalgae, were run in parallel with the experiments to assess potential losses due to sorption onto glass vessel walls or during the centrifugation process. Blanks, defined as macroalgae with only saline water without contaminants, were also performed.

### Data analysis

The removal efficiency (*R*, %) of the chemical elements was calculated based on their initial concentration ($C_{0}$, µg L^− 1^) and concentration at the time $t$ ($C_{t}$, µg L^− 1^) in the solution by using the following equation [27]:

1 $$R = \frac{{\left( {C_{0} - C_{{\mathrm{t}}} } \right)}}{{C_{0} }} \times 100$$

Considering that all elements removed from the solution were taken up by the macroalgae, the mass of elements per unit mass of seaweed at the end of the experiment ($q_{{\mathrm{t}}}$, µg g^− 1^) was estimated by the time $t$ ($C_{t}$, µg L^− 1^) in the solution by using the following equation [27]:

2 $$q_{{\mathrm{t}}} = \frac{{\left( {C_{0} - C_{{\mathrm{f}}} } \right) \times V}}{m}$$

where $C_{{\mathrm{f}}}$ is the element’s final concentration in the solution (µg L^− 1^), *m* is the mass of macroalgae (g dry weight basis), and $V$ is the solution volume (L). $C_{0}$ has the same meaning as in Eq. (1).

The bioconcentration factor (BCF, L g^− 1^), being defined as the ratio between an element concentration in the macroalgae (*q*_*real*_, µg g^− 1^) and the initial concentration in saline solutions ($C_{0}$, µg L^− 1^), was calculated as follows [29]:

3 $$BCF = \frac{{q_{real } \times 1000}}{{C_{0} }}$$

The uptake kinetics of the sorption by living and non-living macroalgal biomass were studied by the adjustment of the kinetic models, Lagergren’s pseudo-first-order model (Eq. 4), Ho’s pseudo-second order model (Eq. 5), and Elovich’s model (Eq. 6) [34] to the experimental data:

4 $$q_{t} = q_{e} \left( {1 - e^{{k_{1} t}} } \right)$$

5 $$q_{t} = \frac{{q_{e}^{2} k_{2} t}}{{1 + q_{e} k_{2} t}}$$

6 $$q_{t} = \frac{1}{\beta }\ln \left( {1 + \alpha \beta t} \right)$$

where *q*_*t*_ is the concentration of the chemical element in the biomass at time *t*, calculated by Eq. 2 through the replacement of *C*_*f*_ by the concentration of the chemical element in solution at time *t* (C_f_, µg L^− 1^), *q*_*e*_ is the amount of element bound by unit of mass when the equilibrium is attained (µg g^− 1^), *k*_*1*_ is the rate constant of pseudo first-order model (h^− 1^), k_2_ is the rate constant of pseudo-second-order model (g µg^− 1^ h^− 1^), *α* is the initial sorption rate at zero coverage (µg g^− 1^ h^− 1^), and *β* is the desorption constant (g µg^− 1^). Model-fitting was achieved using GraphPad Prism 6 software. Statistical analysis was limited to descriptive statistics, including calculation of means ± standard deviations and evaluation of temporal trends; no inferential or significance tests were applied.

## Results

### Removal percentages for living and non-living macroalgal biomass

**Fig. 2:**
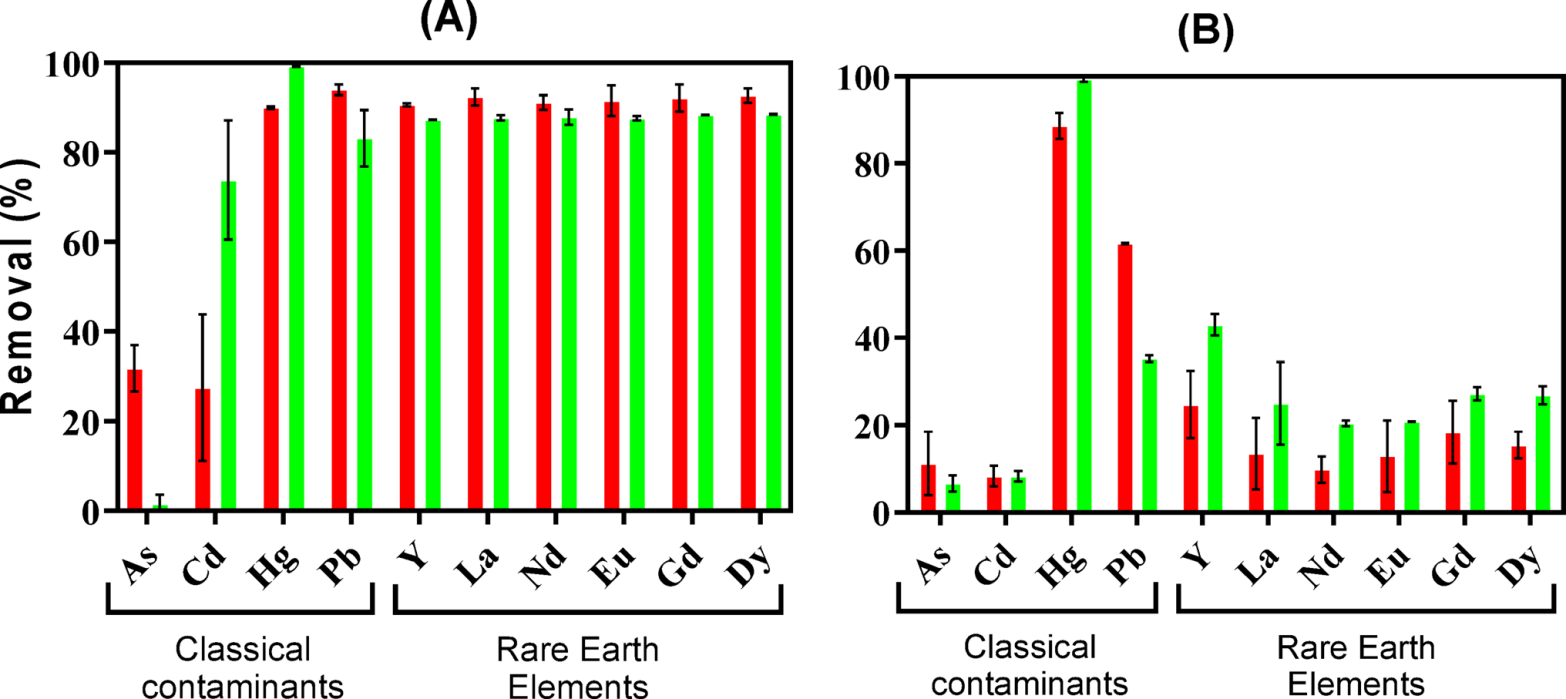
Comparison of the removal percentages for each element after 72 h exposure using living (**A**) and non-living (**B**) biomass of the macroalgae *U. lactuca* (green) and *G. gracilis* (red). Error bars represent the standard deviation of the mean from replicate experiments (n = 2), ranging from 0.06 to 16 for living biomass and from 0.2 to 9.4 for non-living biomass

Figure 2A presents the mean removal percentages (n = 2) obtained after 72 h exposure for living biomass from *U. lactuca* (light green bars) and *G. gracilis* (light red bars) to a multi-contaminant mixture of As, Cd, Y, La, Nd, Eu, Gd, Dy, Hg, and Pb in equimolar concentrations. Removal rates for REEs were over 80%, with values being similar between the two macroalgae tested. For the elements As and Cd, the removal percentages were notably lower (below 40%) except for *U. lactuca* for the element Cd. As for Hg and Pb, respectively, the removals achieved were above 90.0% and 83.2%.

Figure 2B shows the mean removal percentages (n = 2) obtained when exposing non-living biomass of *U. lactuca* (dark green bars) and *G. gracilis* (dark red bars) to the same multi-contaminant mixture, after 72 h of exposure. Compared to the living biomass, the removal percentages achieved for the REEs decreased substantially and were, in general, below 40%, except for *U. lactuca* for the element Cd. Only for the element Hg, the performance of the non-living biomass was maintained and achieved removal percentages similar to the living biomass (above 88.6%). As for the elements Pb, As, and Cd, the removal percentages were even lower than for the living biomass, less than 61.6%, 11.2% and 8.3%, respectively.

### Removal kinetics for living and non-living macroalgal biomass

**Fig. 3:**
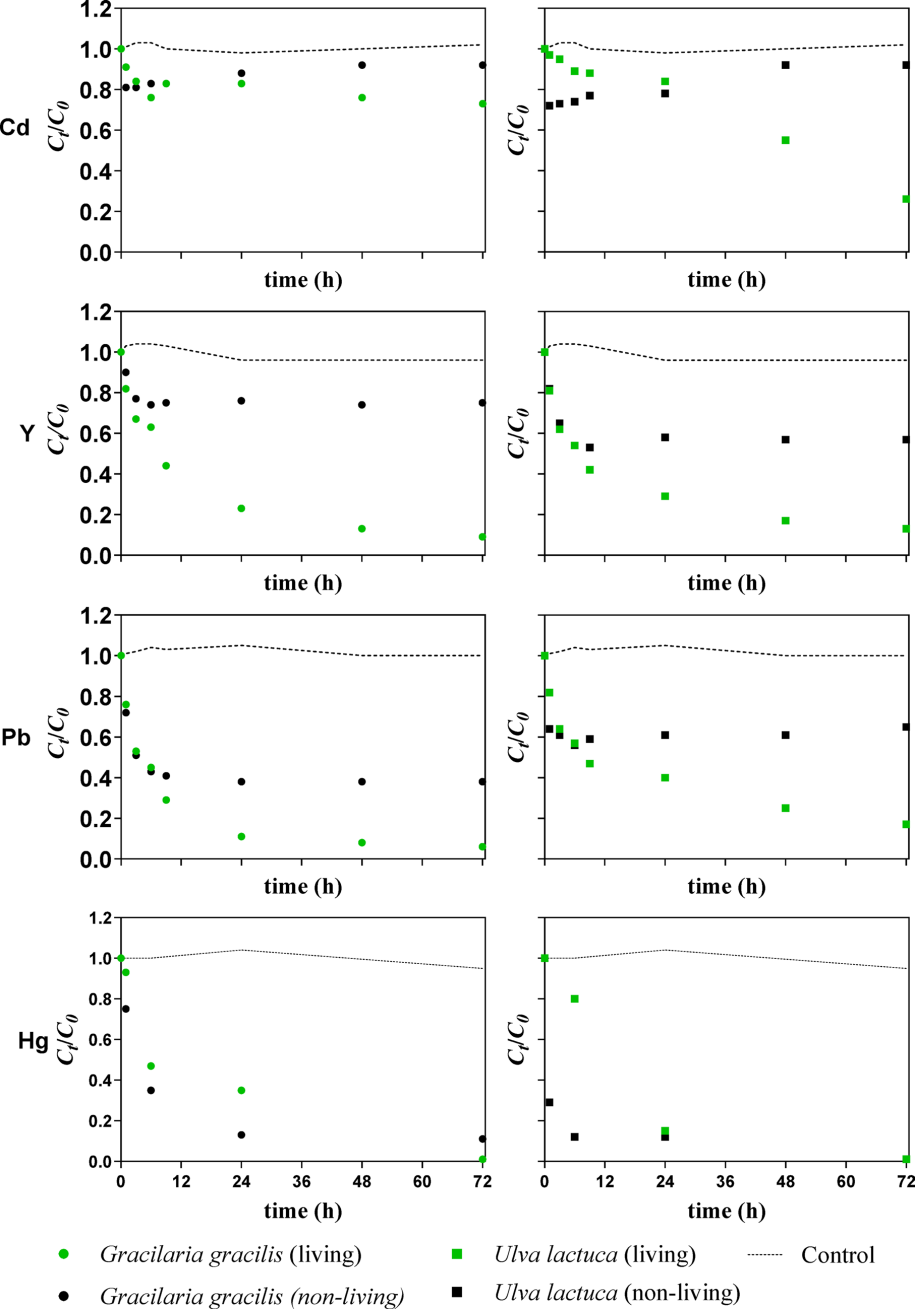
Normalised concentrations (*C*_*t*_*/C*_*0*_, where *C*_*t*_ represents the element concentration at a given time t, and *C*_*0*_ the initial element concentration) of Cd, Y, Pb, and Hg for *U. lactuca* and *G. gracilis* using living and non-living biomass (n = 2; standard deviations ranging from 0.01 to 0.18). The dashed line indicates the concentration of the elements in the solution over 72 h in the absence of any sorbent (control).

Figure 3 exhibits the removal kinetic profiles of Cd, Y, Pb, and Hg—selected as representative elements of distinct contaminant groups and removal behaviours observed in this study—from the mixture by living and non-living biomass of *G. gracilis* (graphs on the left) and *U. lactuca* (graphs on the right), for an exposure of up to 72 h.

Cadmium removal was globally low for both macroalgae in living and non-living forms, except for *U. lactuca* living biomass, for which the removal kinetics increased to almost 80% between 24 and 72 h of exposure. Cd removal occurred mainly in less than 12 h, except for *U. lactuca* living biomass.

The elements Y and Pb were removed more intensively from the solutions by the living biomass of both macroalgae, with removal rates of over 85% after 72 h of exposure. Even for the living and non-living biomasses of both macroalgae, the removal kinetics of Y and Pb followed similar trends in the first 6 h of exposure. The living biomass of both macroalgae was able to further sorb these two metals after this exposure period, while the non-living biomass of both macroalgae did not remove additional amounts of these metals until the end of the experiments.

As for Hg, this element was removed from the solution more quickly by the non-living biomass of both macroalgae, especially by *U. lactuca*, which had a removal rate of almost 85%, after 6 h of exposure. Although the living biomass of *G. gracilis* and *U. lactuca* achieved similar or higher (in the case of *G. gracilis*) Hg removal rates than the non-living biomasses at 72 h of exposure, at 6 h, the removal rates were higher for the non-living biomass of both macroalgae.

### Bioconcentration factors in macroalgal biomass

**Table 1:** Bioconcentration factors in macroalgal biomass (living and non-living) of *U. lactuca* and *G. gracilis*, after 72 h of exposure to the multi-element saline solution

	Species	Element	Initial concentration in water (C_0_, µg L^− 1^)	Initial concentration in water (C_0_, µM)	Initial concentration of biomass (µg g^− 1^)	Theoretical *qt* (µg g^− 1^)	Bioconcentration factor (BCF) (L g^− 1^)
Living biomass	*G. gracilis*	As	110	1.47	6.2	58.3 ± 9.43	530
		Cd	100	0.89	0.29	45.8 ± 27.1	458
		Y	55	0.61	0.6	82.3 ± 5.66	1511
		La	120	0.86	< 1.3	185 ± 25.6	1541
		Nd	120	0.83	< 1.3	183 ± 24.8	1521
		Eu	135	0.89	< 1.3	207 ± 40.1	1531
		Gd	130	0.83	< 1.3	200 ± 28.3	1538
		Dy	140	0.86	< 1.3	216 ± 25.6	1546
		Hg	323	1.61	0.015	342 ± 65.8	1059
		Pb	165	0.80	26	259 ± 14.5	1567
	*U. lactuca*	As	100	1.33	6.2	1.76 ± 2.5	18
		Cd	100	0.89	0.19	86.8 ± 31.3	868
		Y	54	0.61	0.4	55.5 ± 1.41	1027
		La	110	0.79	< 1.3	114 ± 0.83	1032
		Nd	115	0.80	< 1.3	119 ± 4.99	1033
		Eu	125	0.82	< 1.3	129 ± 5.82	1031
		Gd	125	0.79	< 1.3	130 ± 7.49	1040
		Dy	135	0.83	< 1.3	141 ± 7.49	1041
		Hg	160	0.80	0.015	187 ± 35.7	1168
		Pb	165	0.80	5.2	162 ± 19.1	980
Non-living biomass	*G. gracilis*	As	105	1.39	–	20.0 ± 14.1	191
		Cd	120	1.07	–	16.7 ± 4.71	139
		Y	90	1.01	–	37.5 ± 15.3	419
		La	140	1.01	–	32.5 ± 22.4	232
		Nd	140	0.97	–	23.3 ± 9.43	167
		Eu	150	0.99	–	33.3 ± 23.6	222
		Gd	160	1.02	–	50.0 ± 23.6	313
		Dy	160	0.98	–	41.7 ± 11.8	260
		Hg	221	1.10	–	328 ± 81.2	1483
		Pb	215	1.04	–	221 ± 22.4	1027
	*U. lactuca*	As	97	1.29	–	7.65 ± 2.50	79
		Cd	120	1.07	–	11.8 ± 1.66	98
		Y	78	0.88	–	39.4 ± 0.83	505
		La	120	0.86	–	35.3 ± 13.3	294
		Nd	115	0.80	–	27.7 ± 0.83	240
		Eu	115	0.76	–	28.2 ± 1.66	246
		Gd	125	0.79	–	40.0 ± 0.00	320
		Dy	125	0.77	–	39.4 ± 0.83	315
		Hg	205	1.02	–	240 ± 44.0	1169
		Pb	180	0.87	–	74.7 ± 7.49	415

Table 1 shows the basal concentrations in the macroalgal biomass and the calculated bioconcentration factors (BCF) (n = 2) for living and non-living biomass of *U. lactuca* and *G. gracilis* after 72 h of exposure to the multi-element saline solution.

The BCF values show high variability between 18 and 1567 L g^− 1^ for living biomass, and 79 and 1483 L g^− 1^ for non-living biomass. Generally, BCF values are lower for As and Cd and higher for lanthanides.

### Sorption kinetics

**Fig. 4:**
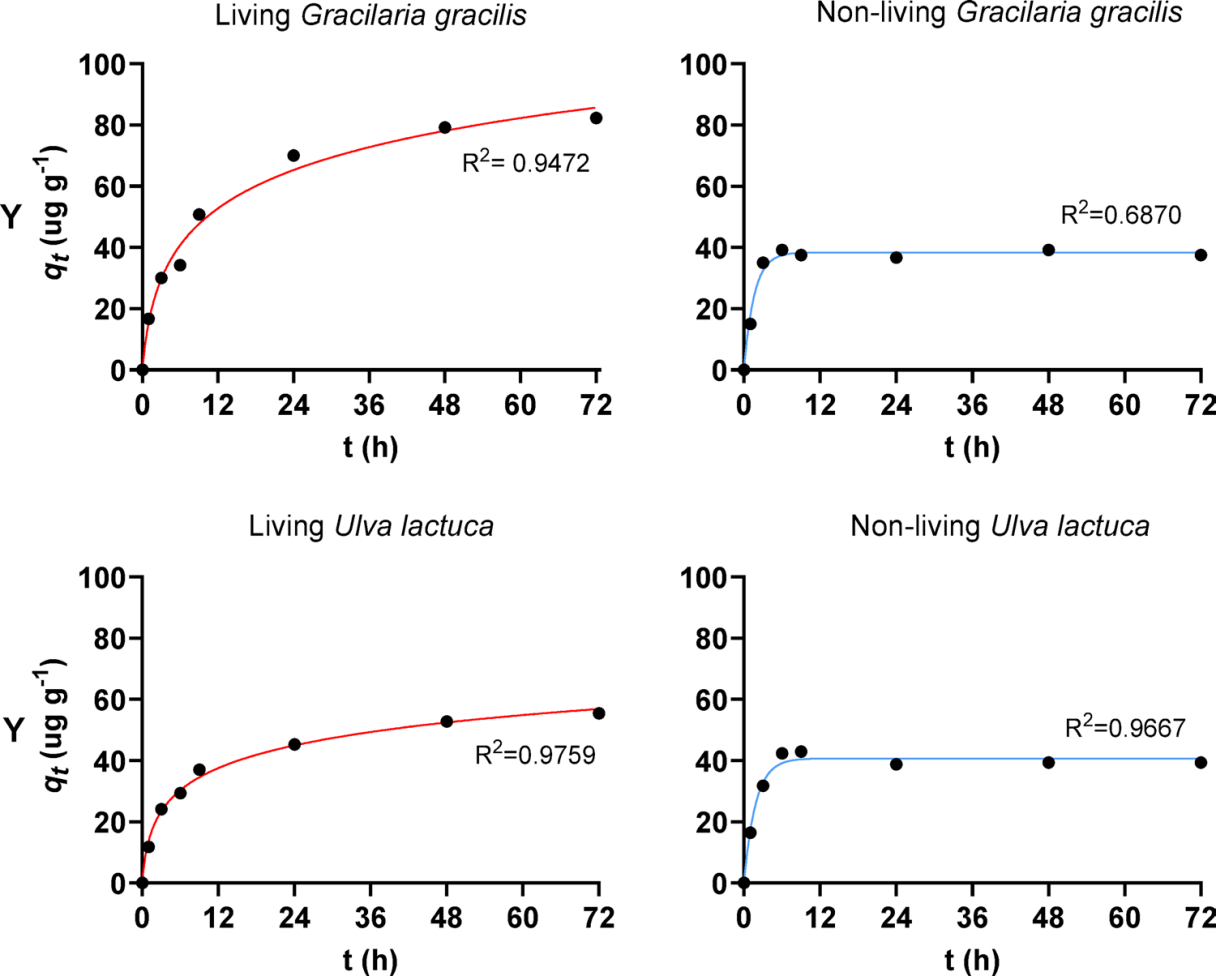
Fittings of Elovich (in red) and pseudo-first order (in blue) models to experimental data on Y uptake by living and non-living macroalgal biomass.

Figure 4 shows the kinetic modelling curves that best fit the sorption of Y by living and non-living macroalgal biomass. The data for the remaining elements can be found in Figure S1 (supplementary material). The fitting based on three kinetic models, namely pseudo-first order, pseudo-second order and Elovich, generally yielded good fits to the experimental data for all elements and for both living and non-living biomass. The coefficient of determination (R^2^) varied between 0.6065 and 0.9950, except for the non-living biomass of the red macroalgae *Gracilaria gracilis* for the elements Y, La, Nd, Eu, and Gd and for the non-living biomass of the green macroalgae *U. lactuca* for Eu and Nd. Tables S1, S2, S3 and S4 (supplementary material) summarise the best-fit parameters and goodness of fit for living and non-living biomass of both macroalgae. Globally, living biomass (both red and green) is characterised by a fast and marked uptake of elements in the first few hours, followed by a less pronounced increase in element content in the biomass, with no major differences between REEs profiles. The uptake of Pb and Hg was even faster in the first hours. The models that best suited the uptake of REEs, Pb, and Hg varied; therefore, there was no consensus as to which model showed the most effective performance in describing the uptake kinetics during the studied period. The kinetic profiles of living and non-living biomass show the greatest differences for REEs. The uptake by the non-living biomass occurs very quickly and in fewer hours than by the living biomass. A plateau was attained solely for the non-living biomass, indicating that the uptake for the living biomass could potentially extend further with longer exposure times. Furthermore, the process reached equilibrium more rapidly for the non-living biomass. For the elements Pb and Hg, the kinetic profiles of element uptake by living and non-living macroalgal biomass were more similar. However, like REEs, uptake is faster and reaches equilibrium more quickly in the non-living biomass.

## Discussion

The sorption behaviour and underlying mechanisms of macroalgae depend strongly on species-specific chemical composition and biological traits, as well as on the mode of application (living *vs*. non-living), the presence of competing ions, and the affinity of the sorbent for the different types of contaminants. Despite increasing interest in macroalgae-based sorption, significant gaps remain in understanding how these parameters interact in complex mixtures. The present work addresses several of these gaps by comparatively evaluating red and green macroalgae, in both living and non-living biomass, exposed to a chemically complex saline solution containing REEs and classical contaminants.

### Differences among macroalgae species

When comparing macroalgae species, morphological features such as the external surface area of the biomass are often considered determinants of sorption efficiency [46]. Previous studies attributed the superior performance of living biomass of *U. lactuca* to its flat, sheet-like thallus with two structurally uniform cell layers that provide a large surface area, maximising the accessibility of binding sites [28, 29, 55]. In the present work, although no major overall differences were observed between *U. lactuca* and *G. gracilis*, species-specific affinities emerged depending on the element and biomass condition. Living biomass of *U. lactuca* showed a superior performance for Cd and Hg, while *G. gracilis* performed better for As (Fig. 2A). The non-living biomass of *U. lactuca* (Fig. 2B) showed higher affinity for Y, Dy and Hg, while the non-living biomass of *G. gracilis* achieved a higher Pb removal.

These findings are consistent with previous work by Pinto et al. [47], who reported high REEs removal efficiencies for *U. lactuca* and comparable performance for *G. gracilis* under mono-element conditions, suggesting that external contact area does not fully explain sorption behaviour. Instead, the chemical composition of the macroalgal cell walls likely plays an important role. Macroalgae cell walls contain a variety of functional groups, such as amino, hydroxyl, carbonyl, and sulphate groups, which act as binding sites via ion exchange, electrostatic attraction, and surface complexation [6, 12, 32, 48]. These negatively charged groups exhibit high affinity for trivalent cations such as REEs and Pb^2+^, explaining their rapid initial uptake in both living and non-living biomass.

The nature and abundance of these functional groups differ among algal taxa due to their dominant structural polysaccharides [51]. Green macroalgae (Chlorophyta), such as *U. lactuca*, are rich in ulvan, while red macroalgae (Rhodophyta), such as *G. gracillis*, contain sulphated galactans, including agar and carrageenans (Martins et al., 2023). *Gracilaria* species are agarophytes that produce highly sulphated polysaccharides, providing a high density of negatively charged sites that favour cation complexation (Yahyaoui et al., 2024) [58]. This chemical differentiation likely explains the observed selectivity patterns, particularly for Pb in *G. gracillis*.

Selective binding preferences reported in the literature further support this interpretation. Son et al. [51] demonstrated that carboxylic groups preferentially bind divalent cations such as Pb^2+^, while amine groups showed greater affinity for Hg^2+^. For ulvan-rich biomass, the reported metal affinity sequence (Pb > Zn > Cd = Mn > Sr > Mg = Ca) (Kumar et al., 2016) aligns well with the higher Pb removal observed for *U. lactuca*. Therefore, the combined influence of surface area and species-specific functional group chemistry provides a mechanistic explanation for the element- and species-dependent sorption patterns observed.

Although not directly evaluated in this study, species-specific tolerance to the specific properties of the wastewater (such as concentration and type of contaminants present, salinity and pH) may also influence sorption performance by influencing cell wall integrity, surface charge, and metabolic activity [5, 16, 56].

### Differences among elements

In multi-element systems, sorption behaviour reflects a balance between intrinsic metal properties, solution chemistry, and competition for available binding sites [36, 43]. In the present study, living macroalgal biomass (regardless of the species) exhibited the highest affinity for Hg (99.3%) followed by Pb (94.0%) and REEs (> 80%), while the classical contaminants As (31.8%) and Cd (27.5%) were less removed from solution (showing similar kinetic profiles that differed from the other elements—Fig. 3). This behaviour is consistent with the literature. The exceptional affinity for Hg is well documented and can be attributed to its strong interaction with sulphur- and nitrogen-containing functional groups, as well as its favourable redox chemistry, which favours stable binding to biological materials regardless of metabolic activity [10, 23]. Importantly, Hg sorption appears largely insensitive to competition from other cations, explaining its consistently high removal even in complex mixtures. In contrast, Cd exhibited lower affinity, which can be rationalised by its lower covalent index, smaller ionic radius and lower electronegativity relative to Pb and Hg, reducing its tendency to form stable complexes with biological ligands [24, 42].

The low removal efficiency observed for As is likely related to its aqueous speciation under the studied conditions. At near-neutral pH, As predominantly exists as negatively charged or neutral oxyanions, which exhibit weak electrostatic attraction to the negatively charged macroalgal surface [35]. Consequently, Cd and As are more strongly affected by competition with REEs and Pb, which explains their consistently lower removal percentages.

REEs sorption behaviour reflects both their chemical similarity and their sensitivity to solution chemistry. In saline environments, REEs, can form complexes with sulphate, carbonate, and dissolved organic matter (e.g., REE^3+^, REE(SO_4_)^+^, REE(CO_3_)^+^ and REE-DOC), with speciation controlled by pH, redox conditions, and ligand availability [40], Banaee et al., (2025). Competition with major cations such as Ca^2+^ and Mg^2+^ may further reduce bioavailability by interfering with the occupation of binding sites or altering surface charge (Gong et al., 2020). Despite these constraints, high REEs removal efficiencies were observed, indicating a strong affinity of macroalgal functional groups for trivalent cations.

Kinetics profiles (Figs. 3 and 4) further support these mechanistic interpretations. Yttrium (and the lanthanides (not shown) that followed a similar *C*_*t*_*/C*_*0*_ profile, explained by their chemical similarities) displayed an accentuated decrease in the first six hours (Fig. 3), followed by a plateau, particularly for non-living biomass, consistent with fast surface-controlled sorption processes. This behaviour can be attributed to the sorbent initially having more sites available to interact with the ions, corresponding to a faster decline in their concentration in solution over this period [54]. In contrast, living biomass showed continued uptake beyond 24 h, suggesting additional mechanisms beyond surface binding. Lead exhibited rapid initial removal followed by saturation, while Hg showed the steepest decline in concentration within the first 24 h, reflecting its exceptional binding affinity. Overall, the affinity sequence observed (Hg > REEs and Pb > As and Cd) highlights the dominant role of chemistry and competitive interactions in controlling sorption outcomes. From an application perspective, the efficient and rapid removal of priority contaminants such as Hg and Pb, as well as emerging contaminants like REEs, even in a chemically complex matrix, is highly advantageous for water treatment. However, pre-treatment steps might still be needed before exposing the macroalgae to real effluents, since some characteristics, such as variable pH and organic matter content, may further influence sorption performance. Despite the duplicate experimental runs per treatment (n = 2), the obtained results show very consistent trends, and it is possible to observe large effect sizes, indicating that the experimental findings remain meaningful.

### Living *versus* Non-living biomass

The comparison between living and non-living biomass is a key contribution of the present work, which is underexplored in the literature. While both biomass types were able to efficiently uptake Hg and Pb, marked differences were observed for REEs. Non-living biomass relied exclusively on physicochemical mechanisms, such as ion exchange, complexation, and electrostatic interactions—leading to rapid site saturation and limited REEs uptake [15, 41]. In contrast, living biomass consistently achieved higher REEs removal, even in the presence of competing contaminants. The enhanced performance strongly suggests the involvement of biologically mediated processes [12, 48]. In living macroalgal biomass, elements may be adsorbed onto the cell surface, transported across the cell wall, or accumulated intracellularly, as documented for both nutrients and toxic metals [15, 20]. The continued uptake observed beyond the equilibrium phase of non-living biomass supports the hypothesis that metabolic activity, biomass growth, and the generation of new binding sites contribute to sustained sorption [3]. Differences in kinetic model fitting between living and non-living biomass (Fig. 4) further reinforce this interpretation.

When comparing sorption processes with different biosorbents, time is a crucial factor, since shorter times are preferable from the economic/industrial point of view. From that perspective, the use of non-living materials, which typically operate with shorter contact times, offers an advantage [9]. Importantly, the contrasting selectivity of living and non-living biomass opens opportunities for process optimisation. For instance, a sequential approach could exploit the strong affinity of non-living biomass for Hg, allowing REEs to remain in solution for subsequent recovery using living biomass. Such strategies could enhance selectivity while maintaining sustainability.

Finally, regeneration of the biosorbent is critical for economic feasibility. Previous studies indicate that macroalgal biomass can be regenerated using relatively mild treatments, suggesting that repeated use is achievable. By strategically combining living and non-living biomass, macroalgae-based technologies can be tailored to balance selectivity, efficiency, and sustainability in complex saline waters.

## Conclusions and future perspectives

Macroalgae, regardless of the species, present the highest sorption efficiency for the classical contaminants Hg (99.3%) and Pb (above 80%), followed by the REEs (numerically identical—92.7%), while the classical contaminants As and Cd are less removed (< 40%). However, the main findings of this study were the different affinities observed for REEs between living and non-living biomass. Non-living biomass showed less affinity for these metals while maintaining it for Hg.

From the perspective of improving water quality, the living biomass would be the most effective to achieve the removal of various contaminants, being a readily available feedstock with no need for pre-treatment. Nevertheless, the lack of selectivity of the living macroalgal biomass can be a disadvantage when attempting to isolate economically valuable elements. This could be overcome with a prior step of applying the non-living biomass to remove Hg and other contaminants. A combination of the two forms of the sorbent (living and non-living biomasses) is proposed to simultaneously improve water quality and recover REEs.

From the perspective of industrial application, when compared to conventional sorbents, living macroalgal biomass has a much simpler implementation, not requiring synthesis and/or processing pre-treatments. Furthermore, they are easily harvested from the solution, which may guarantee lower costs associated with this step. Living macroalgal biomass offers the possibility of sorbent regeneration, representing an advantage over non-living biomass, allowing a potential industrial application in continuous systems. Non-living macroalgal biomass does not require any chemical activation, and its obtention comes from an abundant source, presenting low implementation needs. After the sorption process, these sorbents might undergo a pyrolysis step for the recovery of the REEs, after which electrochemical techniques should be applied to separate the different elements. This step works as a pre-concentration method for these elements. Possible scale-up of the technology would involve a cultivation step of the biosorbent. Future studies should evaluate techno-economic feasibility and life-cycle impact of combined living/non-living macroalgal biomass systems.

## Supplementary Information

Below is the link to the electronic supplementary material.

Supplementary Material 1.

## Data Availability

The authors declare that the data supporting the findings of this study are available within the paper and its Supplementary Information files. Should any raw data files be needed in another format, they are available from the corresponding author upon reasonable request.
