# Supplementary material for: Comparing the efficiency of living and non-living macroalgae biomass in removing classical and emergent contaminants from complex multi-element mixtures
Source: Bioprocess Biosyst Eng. 2026 Feb 25;49(4):877–89. doi: 10.1007/s00449-026-03292-z (PMC13124917; doi:10.1007/s00449-026-03292-z)
Supplement: Supplementary file 1 — Supplementary Material 1. [file 449_2026_3292_MOESM1_ESM.docx]

**Comparing the efficiency of living and non-living macroalgae biomass in removing classical and emergent contaminants from complex multi-element mixtures**

Supplementary Material Section

In this section, Figure S1 and Tables S1, S2, S3 and S4 are presented, which support and complement the information presented in the article.

**Figure S1** – Fittings of pseudo-first order (in blue), pseudo-second order (in green) and Elovich (in red) models to experimental data on Y, Dy, Pb and Hg uptake by living and non-living macroalgal biomass.

**Table S1, 2,3 and 4** – Parameters of the fittings of pseudo-first order, pseudo-second-order and Elovich models to experimental data on Y, Dy, Pb and Hg uptake by living and non-living macroalgal biomass.

**Figure S1** – Fittings of pseudo-first order (in blue), pseudo-second order (in green) and Elovich (in red) models to experimental data on Y, Dy, Pb and Hg uptake by living and non-living macroalgal biomass.

**Table S1**– Parameters of the fittings of pseudo-first order, pseudo-second order and Elovich models to experimental data on Y, Dy, Pb and Hg, respectively, uptake by living biomass of *Ulva lactuca*.

|  | Living biomass – *Ulva lactuca* | | | | | | | | |
| --- | --- | --- | --- | --- | --- | --- | --- | --- | --- |
|  |  | Y | La | Nd | Eu | Gd | Dy | Pb | Hg |
| Pseudo-first order | qe ±SD (µg g-^1^) | 51.43 | 107.7 | 116.2 | 126.5 | 126.5 | 134.7 | 141.8 | 211.3 |
|  | k_1_±SD (h^-1^) | 0.1590 | 0.1116 | 0.1018 | 0.09534 | 0.1094 | 0.1193 | 0.1677 | 0.1040 |
|  | R^2^ | 0.9475 | 0.9626 | 0.9699 | 0.9697 | 0.9742 | 0.9709 | 0.8905 | 0.9919 |
|  | Sy.x | 4.533 | 8.233 | 7.900 | 8.644 | 7.993 | 9.045 | 18.38 | 9.185 |
| Pseudo-second order | qe ±SD (µg g-^1^) | 57.37 | 124.4 | 135.1 | 148.2 | 146.0 | 145.2 | 158.7 | 245.0 |
|  | k_2_±SD (h^-1^) | 0.0036 | 0.001039 | 0.0008622 | 0.0007202 | 0.0008697 | 0.0009205 | 0.001351 | 0.0004359 |
|  | R^2^ | 0.9723 | 0.9737 | 0.9734 | 0.9719 | 0.9784 | 0.9807 | 0.9295 | 0.9950 |
|  | Sy.x | 3.291 | 6.911 | 7.426 | 8.335 | 7.325 | 7.371 | 14.75 | 7.249 |
| Elovich | β ±SD (g µg-^1^) | 0.09141 | 0.03677 | 0.03338 | 0.02968 | 0.03133 | 0.03042 | 0.03329 | 0.01723 |
|  | α ±SD (µg g^-1^ h^-1^) | 27.38 | 28.09 | 27.01 | 26.09 | 32.40 | 39.81 | 80.61 | 36.64 |
|  | R^2^ | 0.9759 | 0.9700 | 0.9666 | 0.9650 | 0.9695 | 0.9742 | 0.9493 | 0.9924 |
|  | Sy.x | 3.072 | 7.377 | 8.315 | 9.297 | 8.700 | 8.524 | 12.50 | 8.872 |

**Table S2**– Parameters of the fittings of pseudo-first order, pseudo-second order and Elovich models to experimental data on Y, Dy, Pb and Hg, respectively, uptake by living biomass of *Gracilaria gracilis*.

|  | Living biomass – *Gracilaria gracilis* | | | | | | | | |
| --- | --- | --- | --- | --- | --- | --- | --- | --- | --- |
|  |  | Y | La | Nd | Eu | Gd | Dy | Pb | Hg |
| Pseudo-first order | qe ±SD (µg g-^1^) | 79.14 | 184.9 | 193.4 | 216.2 | 204.3 | 216.7 | 249.6 | 332.5 |
|  | k_1_±SD (h^-1^) | 0.1146 | 0.06372 | 0.04382 | 0.04501 | 0.05328 | 0.06118 | 0.1919 | 0.2138 |
|  | R^2^ | 0.9353 | 0.8734 | 0.8771 | 0.8355 | 0.8825 | 0.9061 | 0.9636 | 0.7959 |
|  | Sy.x | 7.839 | 26.89 | 27.32 | 35.70 | 28.10 | 26.42 | 18.04 | 82.23 |
| Pseudo-second order | qe ±SD (µg g-^1^) | 90.54 | 231.4 | 263.7 | 291.1 | 264.6 | 272.6 | 272.4 | 381.7 |
|  | k_2_±SD (h^-1^) | 0.001520 | 0.0002651 | 0.0001350 | 0.0001291 | 0.0001811 | 0.0002148 | 0.001002 | 0.0005684 |
|  | R^2^ | 0.9468 | 0.8714 | 0.8718 | 0.8320 | 0.8795 | 0.9054 | 0.9800 | 0.8018 |
|  | Sy.x | 7.113 | 27.11 | 27.90 | 36.08 | 28.46 | 26.52 | 13.37 | 81.04 |
| Elovich | β ±SD (g µg^-1^) | 0.05290 | 0.01629 | 0.01201 | 0.01117 | 0.01327 | 0.01368 | 0.02099 | 0.01244 |
|  | α ±SD (µg g^-1^ h^-1^) | 24.15 | 19.10 | 10.66 | 12.75 | 15.86 | 21.26 | 213.9 | 133.6 |
|  | R^2^ | 0.9472 | 0.8661 | 0.8653 | 0.8270 | 0.8744 | 0.9013 | 0.9670 | 0.7881 |
|  | Sy.x | 7.083 | 27.66 | 28.61 | 36.61 | 29.05 | 27.09 | 17.18 | 83.79 |

**Table S3** – Parameters of the fittings of pseudo-first order, pseudo-second order and Elovich models to experimental data on Y, Dy, Pb and Hg, respectively, uptake by non-living and non-living biomass of *Ulva lactuca*.

|  | Non-living biomass – *Ulva lactuca* | | | | | | | | |
| --- | --- | --- | --- | --- | --- | --- | --- | --- | --- |
|  |  | Y | La | Nd | Eu | Gd | Dy | Pb | Hg |
| Pseudo-first order | qe ±SD (µg g-^1^) | 40.57 | 36.16 | 27.53 | 29.37 | 41.00 | 40.98 | 85.32 | 229.6 |
|  | k_1_±SD (h^-1^) | 0.5502 | 0.2779 | 0.1726 | 0.1311 | 0.1698 | 0.2004 | 2.255 | 1.384 |
|  | R^2^ | 0.9667 | 0.8387 | 0.6457 | 0.6175 | 0.7328 | 0.7588 | 0.8936 | 0.8902 |
|  | Sy.x | 2.806 | 6.551 | 10.46 | 12.11 | 12.20 | 10.88 | 10.31 | 30.15 |
| Pseudo-second order | qe ±SD (µg g-^1^) | 42.51 | 39.07 | 31.68 | 34.94 | 47.02 | 46.09 | 85.99 | 236.8 |
|  | k_2_±SD (h^-1^) | 0.02258 | 0.009865 | 0.005498 | 0.003478 | 0.003747 | 0.004875 | 0.1155 | 0.01037 |
|  | R^2^ | 0.9228 | 0.7766 | 0.6065 | 0.5860 | 0.6895 | 0.7097 | 0.8908 | 0.8999 |
|  | Sy.x | 4.269 | 7.710 | 11.04 | 12.60 | 13.15 | 11.93 | 10.44 | 28.80 |
| Elovich | β ±SD (g µg^-1^) | 0.2415 | 0.1597 | 0.1336 | 0.1094 | 0.09217 | 0.09958 | 1.850 | 0.06100 |
|  | α ±SD (µg g^-1^ h^-1^) | 2206 | 52.26 | 6.932 | 4.791 | 11.06 | 15.68 | 1,664e+066 | - |
|  | R^2^ | 0.8218 | 0.6781 | 0.5478 | 0.5446 | 0.6260 | 0.6466 | - | 0.9015 |
|  | Sy.x | 6.488 | 9.256 | 11.82 | 13.21 | 14.43 | 13.30 | - | 28.55 |

**Table S3** – Parameters of the fittings of pseudo-first order, pseudo-second order and Elovich models to experimental data on Y, Dy, Pb and Hg, respectively, uptake by non-living and non-living biomass of *Gracilaria gracilis*.

|  | Non-living biomass – *Gracilaria gracilis* | | | | | | | | |
| --- | --- | --- | --- | --- | --- | --- | --- | --- | --- |
|  |  | Y | La | Nd | Eu | Gd | Dy | Pb | Hg |
| Pseudo-first order | qe ±SD (µg g-^1^) | 37.72 | 35.24 | 23.33 | 25.79 | 42.96 | 41.96 | 218.2 | 329.1 |
|  | k_1_±SD (h^-1^) | 0.6416 | 0.5662 | 0.4721 | 0.4263 | 0.3488 | 0.4022 | 0.5659 | 0.2867 |
|  | R^2^ | 0.6870 | 0.4455 | 0.2657 | 0.2188 | 0.3962 | 0.7764 | 0.9486 | 0.8601 |
|  | Sy.x | 12.74 | 15.45 | 17.90 | 20.36 | 22.28 | 9.441 | 18.60 | 53.61 |
| Pseudo-second order | qe ±SD (µg g-^1^) | 39.69 | 37.14 | 24.19 | 27.90 | 46.66 | 44.83 | 232.0 | - |
|  | k_2_±SD (h^-1^) | 0.02719 | 0.02457 | 0.03078 | 0.02052 | 0.009987 | 0.01230 | 0.003912 | - |
|  | R^2^ | 0.6536 | 0.4067 | 0.2049 | 0.2035 | 0.3720 | 0.7483 | 0.9446 | 0.8018 |
|  | Sy.x | 10.04 | 15.98 | 18.58 | 20.56 | 22.72 | 10.02 | 19.32 | 63.81 |
| Elovich | β ±SD (g µg^-1^) | 0.2754 | 0.2684 | 0.3937 | 0.2490 | 0.1362 | 0.1535 | 0.04000 | 0.01921 |
|  | α ±SD (µg g-1 h-1) | 3849 | 1410 | 441.8 | 80.12 | 77.52 | 114.5 | 5603 | 804.1 |
|  | R^2^ | 0.5833 | 0.3421 | 0.1347 | 0.1726 | 0.3218 | 0.6788 | 0.8858 | 0.8018 |
|  | Sy.x | 11.01 | 16.83 | 19.43 | 20.96 | 23.62 | 11.32 | 27.74 | 63.81 |
